# Synthesis and Biological Studies of New Temporin A Analogs Containing Unnatural Amino Acids in Position 7

**DOI:** 10.3390/pharmaceutics16060716

**Published:** 2024-05-27

**Authors:** Dilyana Dimitrova, Veronica Nemska, Tsvetelina Foteva, Ivan Iliev, Nelly Georgieva, Dancho Danalev

**Affiliations:** 1 Biotechnology Department, University of Chemical Technology and Metallurgy, 8 Kliment Ohridski Blvd., 1797 Sofia, Bulgaria; dilyana@uctm.edu (D.D.); vnemska@uctm.edu (V.N.); tsvetelina.foteva@uctm.edu (T.F.); neli@uctm.edu (N.G.); 2 Institute of Experimental Morphology, Pathology and Anthropology with Museum, Bulgarian Academy of Sciences, Acad. G. Bonchev Str., Bl. 25, 1113 Sofia, Bulgaria; taparsky@abv.bg

**Keywords:** antimicrobial resistance, antimicrobial peptides, Temporin A, solid-phase peptide synthesis, antibacterial activity, antiproliferative activity, cytotoxicity, phototoxicity

## Abstract

(1) Background: Antimicrobial resistance is growing at an extreme pace and has proven to be an urgent topic, for research into alternative treatments. Such a prospective possibility is hidden in antimicrobial peptides because of their low to no toxicity, effectiveness at low concentrations, and most importantly their ability to be used for multiple treatments. This work was focused on the study of the effect of the modification in position 7 of Temporin A on its biological activity; (2) Methods: The targeted peptides were synthesized using Fmoc/O*t*-Bu SPPS. The antibacterial activity of the analogs was determined using the broth microdilution method and disk-diffusion method. In vitro tests were performed to determine the cytotoxicity, phototoxicity, and antiproliferative activity of the peptide analogs on a panel of tumor and normal cell lines; (3) Results: All analogs except DTCit showed good antibacterial activity, with DTDab having the best activity according to the disk-diffusion method. However, DTCit had an acceptable cytotoxicity, combined with good selectivity against the test MCF-7 cell line; (4) Conclusions: The obtained results revealed the importance of the basicity and length of the side chain at position 7 in the Temporin A sequence for both tested activities.

## 1. Introduction

The natural mechanisms of microorganisms to ensure their survival in their environment includes the secretion of specific substances able to kill other bacteria and allow the proliferation of the strain. At the same time, these substances must be non-toxic against their strain producer [1]. Most of the antibiotics currently used in medicinal practice are naturally secreted by bacteria or fungi, in order to ensure the process of their survival.

Resistance against the used antimicrobial substances is one of the most prevalent global health threats. The World Health Organization (WHO) stated that a time with untreatable infections with current antibiotics is forthcoming [2]. According to the “Global burden of bacterial antimicrobial resistance” report for 2019, antimicrobial resistance (AMR) was unequivocally accountable for 1.27 million deaths worldwide and indirectly for another 4.95 million. In addition, AMR might surpass cancer mortality in less than 30 years and by 2050 claim 10 million lives annually [3]. 

Antimicrobial peptides (AMPs) have become a promising alternative as broad-spectrum antimicrobial agents [4]. In 1957, for the first time, Robert Skarnes discovered antimicrobial peptides in blood cells. Since then, over 1200 AMPs have been isolated from diverse sources, such as bacteria, plants, insects, and animals [1]. In contrast to conventional antibiotics, AMPs realize contact with the cell membranes by neutralizing the charge, then pass through the membranes to destroy the bacteria and lessen the likelihood that they will develop drug resistance. In addition, they are also effective at low concentrations and on certain types of bacteria that are resistant to common antibiotics such as vancomycin-resistant *Enterococcus* and methicillin-resistant *Staphylococcus aureus*. Furthermore, AMPs can be applied in combination with conventional antibiotics in search of a synergistic effect. Moreover, they are considered safe for use, with less or lack of side effects, and are unlikely to cause drug resistance. As an added benefit, they possess a broad spectrum of antimicrobial properties compared to traditional antibiotics [5,6,7]. Natural peptides have multifunctional activity, i.e., they are able to target several points of interest [1,6]. Most AMPs are cationic, amphiphilic, generally positively charged, and they range from 10 to 60 amino acid residues, with an overall charge of +2 to +9. In addition, they are characterized by the presence of positively charged basic amino acids like lysine and arginine, as well as hydrophobic residues [5,6,8]. 

Although it is challenging to classify natural AMPs, due to their diversity, they can be categorized in several ways:

- According to their origin, AMPs are divided into four groups—mammalian, amphibian, microorganism-derived, and insect-derived. Representatives of the first group include defensins, cathelicidins, and lactoferricin B. A significant part of the amphibian defense against pathogens is provided by own-produced AMPs [9]. The well-known first group magainins are isolated from frogs [10]. Microorganisms such as bacteria and fungi can also yield antimicrobial peptides. Notable examples of such peptides include nisin and gramicidin, which are synthesized by *Bacillus subtilis*, *Lactococcus lactis*, and *Bacillus brevis* strains. The strong ability of insects to adapt is largely due to AMPs, which are primarily synthesized in their blood cells and fat bodies. Found in bees, guppy silkworms, and *Drosophila*, cecropins are the most well-known family of AMPs found in insects [8,11];
- According to their activity—an overview of the activities of AMPs includes peptides with antibacterial, antifungal, antiviral, antiparasitic, and antitumor activities. Antibacterial peptides are a significant part of AMPs and have a comprehensive inhibitory activity against many pathogenic bacteria. Many AMPs such as nisin, defensins, and cecropins have demonstrated good inhibition of Gram-positive and Gram-negative bacteria. Antifungal peptides, a subclass of AMPs, target fungal infections with an increased resistance to drugs. Examples of such peptides include brevinine, cecropin, and ranatuerin. Antiviral peptides are also an important class of AMPs that show a potent killing effect on viruses. Antiparasitic peptides have the ability to eradicate parasites responsible for diseases like malaria and leishmaniasis. Anticancer peptides demonstrate their antiproliferative effects by enlisting immune cells to destroy tumor cells. Thus, cancer cells necrotize or undergo apoptosis. In addition, anticancer peptides block angiogenesis to stop tumor growth as well as metastasis, and lastly activate specific regulatory functional proteins to obstruct tumor cell gene transcription and translation [6,8];
- Based on their structures—there are four groups of AMPs: β-sheet peptides, linear α-helix peptides, and peptides, with and without both structural elements. Hundreds of distinct sequences of the α-helix conformational peptides have already been identified from natural sources. Thus, they represent a relatively diverse and extensively researched class of AMPs. There are many different data in the scientific literature concerning the chain length of the AMPs, but most often they contain 12–40 amino acids, with a high concentration of helix-stabilizing residues like Ala, Lys, and Leu. Every β-family of AMPs possesses a minimum of one pair of two β-strands. Nearly all the AMPs in this group have cysteine residues, which can form one or more disulfide bridges that stabilize their structures. These peptides exhibit greater stability in solution and do not significantly alter the structural makeup of the membrane environment. AMPs with both α-helix and β-sheet elements have been reported in a variety of invertebrates and plants, in addition to humans and other mammals. These AMPs are categorized according to the various configurations of their three to five disulfide bonds. Some AMPs are known as extended linear structures, because they do not take on a particular 3D structure in solution or when they come into contact with membranes. These peptides are typically enriched amino acids, such as Gly, Pro, Trp, or His, and lack α-helices and β-sheets [8,11].

AMPs achieve their antimicrobial effects using two main mechanisms. The membrane-targeting AMPs damage the structure of the cell membrane, whereas the non-membrane-targeting AMPs stop the synthesis of nucleic acids, crucial enzymes, and functional proteins [2,8].

Net charge, amphipathicity, hydrophobicity, membrane curvature, and propensity for self-aggregation are among the physicochemical characteristics of AMPs that play a crucial role in the peptide–membrane interactions that lead to the disruption of membrane integrity. Hydrophobic and cationic interactions are the main types of action of membrane-active AMPs. Initial binding of the positively charged residues of AMPs to the negatively charged bacterial cell surface is realized by electrostatic attraction. Some specific peptide–peptide or lipid–peptide complex structures are formed when the concentration of AMPs adhered to the membrane rises. Once the concentration of AMPs on the membrane has reached a critical level, they enter the core of hydrophobic bilayers and create transmembrane pores within the cytoplasmic membrane. According to the barrel-stave model, AMP molecules self-assemble when they adsorb on the membrane surface through interactions with hydrophilic peptide regions. The peptide bulks rotate perpendicularly to the plasma membrane when the accumulated peptide monomers reach a specific density on the membrane. Lastly, the hydrophilic surface of the channel is directed inward as the peptide bulks form a channel along the hydrophobic portion of the bilayer. The mechanism of action of the toroidal model states that, in contrast to the barrel-stave model, peptides are introduced perpendicularly into the bilayer, to form a peptide–lipid complex rather than peptide–peptide interactions. A “toroidal pore” is formed as a result of the peptide conformation, which encourages a specific membrane curvature that is partially surrounded by phospholipid head groups and partially by peptides. Due to an interaction between the negatively charged polar phospholipid heads and the positively charged cationic peptides, AMPs adhere parallel to the membrane surface in the carpet model. The peptides accumulate to reach a critical concentration, and then reverse their orientation to form micelles with a hydrophobic core within the membranes, which results in membrane dissolution [2,5].

Short, homologous AMPs called temporins were extracted from the skin of frogs belonging to the *Rana genus* by Simmaco et al. [12]. Temporins were labelled as ‘‘*Vespa*-like’’ due to their structural similarity to the short peptides found in the venom of wasps from the genus *Vespa*. Temporins and *Vespa* peptides share certain characteristics, such as hydrophobicity, a low molecular weight, and antimicrobial activity. Leu is the most prevalent amino acid in the sequence of temporins, with hydrophobic residues making up 70% of the peptide [13]. Temporins are the largest group of AMPs found in amphibians. A single specimen can contain up to 10 different isoforms [14]. The most researched peptides of the group as antimicrobials are Temporin A (FLPLIGRVLSGIL-NH_2_, herein abbreviated with DTA), Temporin B (LLPIVGNLLKSLL-NH_2_), Temporin F (FLPLIGKVLSGIL-NH_2_, herein abbreviated with DTF), and Temporin L (FVQWFSKFLGRIL-NH_2_). Their amino acid sequences are primarily made up of hydrophobic residues, and they are C-terminally α-amidated [13]. Most of them are non-toxic to human red blood cells at doses able to kill bacteria [15]. With a net charge of 0 to +3, their activity is especially vectorized to Gram-positive bacteria. This family contains one notable exception, Temporin L, which is extremely effective against both Gram-positive and Gram-negative strains. They are amphipathic α-helical AMPs, to date discovered in nature with a length of 10 to 14 amino acids, making them among the smallest AMPs. Although membrane penetration is not inherently fatal, their mode of action entails changing the permeability of the microbial membrane, which permits both small and large molecules to flow through [14]. The antibacterial activity of Temporin A was found to be significantly influenced by the presence of a hydrophobic residue at the N-terminus and the bulky ones at positions 5 and 12. With mild hemolytic activity, Temporin A is mostly active against Gram-positive bacteria [13]. According to Wade et al., who first reported synthesis of Temporin F, a minor change in position 7 by replacement of a strongly basic Arg residue by less basic Lys residue leads to decreasing the antibacterial activity [15]. Thus, in this study, we continued the work started by Wade et al. to explore the influence of the basicity of the amino acid in the position 7 of the Temporin A molecule on the antibacterial activity, introducing in this position the unnatural amino acids ornithine (Orn), citrulline (Cit), 2,4-diaminobutyric acid (Dab), and 2,3-diaminopropionic acid (Dap). Taking into account that AMPs often have additional antiproliferative activity, due to similar mechanisms of penetration in the cell membrane, we also investigated the phototoxicity, cytotoxicity, and antiproliferative effect of the newly synthesized peptides and compared their activities to those of the parent peptide [16,17].

## 2. Materials and Methods

### 2.1. Synthesis and Analytical Data

The specifically protected amino acids needed for targeted peptide synthesis Fmoc-L-Leu-OH, Fmoc-L-Phe-OH, Fmoc-L-Val-OH, Fmoc-L-Pro-OH, Fmoc-Gly-OH, Fmoc-L-Arg(Pbf)-OH, Fmoc-L-Ile-OH, Fmoc-L-Lys(Boc)-OH, Fmoc-L-Ser(Trt)-OH, Fmoc-L-Cit-OH, Fmoc-L-Orn(Boc)-OH, Fmoc-L-Dab(Boc)-OH, Fmoc-L-Dap(Boc)-OH as well as polymer carrier Fmoc-Rink-Amid-MBHA Resin, activation agents N,N′-Diisopropylcarbodiimide (DIC), N,N,N′,N′-Tetramethyl-O-(1H-benzotriazol-1-yl)uronium hexafluorophosphate (HBTU), 2-(1H-Benzotriazole-1-yl)-1,1,3,3-tetramethylaminium tetrafluoroborate (TBTU), N-Hydroxysuccinimide (HOSu), Benzotriazol-1-yloxy)tripyrrolidinophosphonium hexafluorophosphate (PyBOP), scavenger triisopropylsilane (TIS), base N,N-diisopropylethylamine (DIPEA), and trifluoroacetic acid (TFA) were purchased from Iris Biotech GmbH (Marktredwitz, Germany). The solvents for the synthesis N,N′-dimethylformamide (DMF) and dichloromethane (DCM) were purchased from Valerus Ltd. (Bulgaria). All reagents and solvents were applied without any pretreatment.

For the synthesis of the desired compounds, the established solid-phase peptide synthesis Fmoc(9-fluorenylmethoxycarbonyl)/Ot-Bu strategy on Rink-amide MBHA resin was employed. The amino acids were activated using DIC, HBTU, TBTU, or PyBOP. The coupling reactions were carried out using amino acid/HBTU(or PyBOP or TBTU)/DIPEA/resin at a molar ratio 3/3/3/9/1 or amino acid/DIC/1-HOSu/resin at a molar ratio 3/3/3/1. The deprotection of the Fmoc-group was achieved with a 20% piperidine/DMF solution. The Kaiser test was utilized to monitor both coupling and deprotection reactions. A cocktail of 95% trifluoroacetic acid (TFA), 2.5% triisopropylsilane (TIS), and 2.5% distilled water was used to cleave the final peptides from the resin. Each peptide was extracted as a filtrate in TFA and then precipitated using cold, dry diethyl ether. HPLC-MS/MS analyses were performed after the precipitate had been filtered.

The peptide purity was controlled on a RP-HPLC Agilent Poroshell 120, 100 mm × 4.6 mm column using a Shimadzu LC MS/MS 8045 system, flow rate of 0.30 mL/min, column temperature of 40 °C, and a linear binary gradient, consisting of Mobile phase A: H_2_O (10% Acetonitrile (AcCN); 0.1% HCOOH) and Mobile phase B: AcCN (5% H_2_O; 0.1% HCOOH) with the following scheme over time:
**Time (min)****Mobile phase A (%)****Mobile phase B (%)**0.01802010.0059515.0059515.50802022.008020

The structures of the newly synthesized compounds were established by electrospray ionization mass spectrometry in the SCAN regime/ESI+ mode of ionization, with the following parameters:
Nebulizing gas flow3 L/minHeating gas flow10 L/minInterface temperature350 °CDL temperature200 °CHeat block temperature400 °CDrying gas flow10 L/min

An automated standard polarimeter PolamatA (Carl Zeis, Jena; Anton Paar Opto Tec GmbH, Seelze, Germany) was used to determine optical rotation at c = 1 (MeOH). Melting temperatures were measured using an A. KRÜSS Optronic GmbH semi-automatic melting pointmeter (M3000). Table 1 summarizes all obtained analytical data.

### 2.2. Cell Cultures

The cell lines BALB 3T3 (mouse embryonic fibroblasts) and MCF-12F (human breast epithelial cells) were used as models for healthy tissue. The MCF-7 and MDA-MB-231 cell lines were used as in vitro models for luminal A- and basal B-type breast cancer, respectively. Cell lines were obtained from the American Type Cultures Collection (ATCC, Manassas, VA, USA). Cells were cultured in Dulbecco modified Eagle’s medium (DMEM) supplemented with 10% fetal bovine serum (FBS), 100 U/mL penicillin, and 0.1 mg/mL streptomycin (Sigma-Aldrich, Schnelldorf, Germany) in an incubator at 37 °C, 5% CO_2_ and 95% humidity. Plastic flasks 75 cm^2^ (Biologix, Lenexa, KS, USA) were used to grow the cells.

#### 2.2.1. In Vitro Cytotoxicity Testing (3T3 NRU Test)

The cytotoxicity/phototoxicity testing was performed as described by the OECD Guidelines for the Testing of Chemicals, Section 4, Test No. 432: In Vitro 3T3 NRU Phototoxicity Test. BALB 3T3, clone A31 cells were grown in 75 cm^2^ tissue culture flasks in DMEM high-glucose (4.5 g/L), 10% FBS, and antibiotics at 37.5 °C, in a humidified atmosphere under 5% CO_2_. Cells were plated at 1 × 10^4^ cells / 100 μL culture medium in each well of 96-well microplates and allowed to adhere for 24 h before treatment with test compounds. Peptides were dissolved in DMSO and diluted with culture medium to working concentrations from 4 to 1000 μM. In phototoxicity tests, 96-well plates were irradiated (+Irr) with a dose of 2.4 J/cm^2^ using an artificial solar light simulator Helios-iO (SERIC Ltd., Tokyo, Japan), and the cells were incubated for an additional 24 h. After treatment with a Neutral Red medium, washing, and application of the NR Desorb solution, the absorption was measured on a microplate reader at a wavelength of 540 nm. Cytotoxicity/phototoxicity was expressed as CC_50_ values (concentrations required for 50% cytotoxicity).

#### 2.2.2. In Vitro Antiproliferative Activity

The antiproliferative activity testing was performed on cell cultures using the MTT-dye reduction assay [18]. This assay is based on the metabolism of MTT to insoluble formazan by mitochondrial reductases. The formazan concentration can be determined spectrophotometrically. The measured absorption is an indicator of cell viability. Cell lines (MCF-12F, MCF-7 and MDA-MB-231) were used in the experiments. Cells were plated at a density of 1 × 10^3^ cells/100 µL in each well of 96-well microplates and allowed to adhere for 24 h before treatment with test compounds. A concentration range from 4 to 1000 μM was applied for 72 h. The formazan absorption was registered using a microplate reader at λ = 540 nm. Antiproliferative activities were expressed as IC_50_ values (concentrations required for 50% inhibition of cell growth). 

The statistical analysis included the application of one-way ANOVA followed by Bonferroni’s post hoc test. This test was used to compare the results (IC_50_ values) obtained for all tested peptides and to determine statistically significant differences. *p* < 0.05 was accepted as the lowest level of statistical significance. All results are presented as the mean ± SD.

### 2.3. Antimicrobial Assay

#### 2.3.1. Strains, Media, and Culture Conditions

The parent peptide Temporin A and the novel analogs were subjected to tests against model strains *Escherichia coli* NBIMCC 8785, *Bacillus subtilis* NBIMCC 3562, *Arthrobacter oxydans* NBIMCC 9333, *Pseudomonas aeruginosa* NBIMCC 3700, and *Candida albicans* NBIMCC 74, for the assessment of their antimicrobial activity. The strains were acquired from the National Bank for Industrial Microorganisms and Cell Cultures (NBIMCC, Bulgaria). The strain *E. coli* 8785 was cultured in Luria-Bertani broth (LB, HiMedia, Mumbai, India); *B. subtilis* 3562—in nutrient broth (NB, HiMedia, Mumbai, India); *A. oxydans* 9333; *P. aeruginosa* 3700—in meat peptone agar (MPA) medium; and *C. albicans* 74—in yeast mold (YM) medium. An incubator shaker ES-20/60 (Biosan, Latvia) was used for the cultivation and was set to 30 °C for *B. subtilis* 3562, *A. oxydans* 9333, and *C. albicans* 74; and 37 °C for *E. coli* 8785 and *P. aeruginosa* 3700, with a 24 h shaking period at 120 rpm. The microbial cultures were then adjusted to a turbidity of 0.5 McFarland using a Grant-bio DEN-1B densitometer (Grant Instruments, Royston, UK) for further use.

#### 2.3.2. Disk-Diffusion Method

The disk-diffusion method was used as an additional method, with the aim of determining the percent inhibition of the tested samples against the model strains. Sterile plates with LB/NB/MPA/YM agar medium were inoculated on the surface with 100 μL from each microbial culture and incubated at 30 or 37 °C for 30 min, for the suspension to penetrate the agar. After that, sterile paper discs (HiMedia, Mumbai, India) with a diameter of 6 mm were soaked with 20 µL of the peptide solutions with concentrations of 1.4 mg/mL and 10 mg/mL and placed on the agar surface. Sterile paper discs with 10% EtOH/H_2_O were used as a negative control, and the corresponding strain antibiotic disks (HiMedia, Mumbai, India)—as a positive control. The incubation of the plate was performed at 30/37 °C for 24 h. The antimicrobial activity was analyzed by measuring the diameter of the inhibition zones in mm. The experiments were conducted in triplicates, and the average values for the percent of inhibition as a correlation between the inhibition zone of the peptides over the inhibition zone of the antibiotic multiplied by 100 were calculated.

#### 2.3.3. Determination of Minimal Inhibitory Concentration

The minimal inhibitory concentration (MIC) of the peptides was determined using the broth dilution method. Stock solutions of the peptides with a concentration of 10 mg/mL in 10% EtOH/H_2_O were prepared. Suspensions of the bacteria in the corresponding media were added to 96-well U-shaped bottom polystyrene plates (Deltalab S.L., Spain) and exposed to the peptide in the concentration range from 0 to 320 µg/mL, and a 10% EtOH/H_2_O control, diluted using the same scheme as the peptides, was also used. The plates were then incubated under aerobic conditions at the corresponding temperature for 24 h. The MIC (µg/mL) was assumed as the lowest concentration of the antimicrobial, where there was visible inhibition of the strain. The absorbance was measured at 630 nm (Microplate reader PKL PPC 142, Paramedical srl, Salerno, Italy). To determine the minimal bactericidal concentration (MBC) and minimal fungicidal concentration (MFC), 10 μL from concentrations greater than or equal to the MIC was placed on the corresponding solid nutrient medium. The Petri dishes were then incubated at 30 or 37 °C for 24 h. All experiments were conducted in a triplicate.

## 3. Results

### 3.1. Peptide Synthesis and Characterization

**Figure 1:**
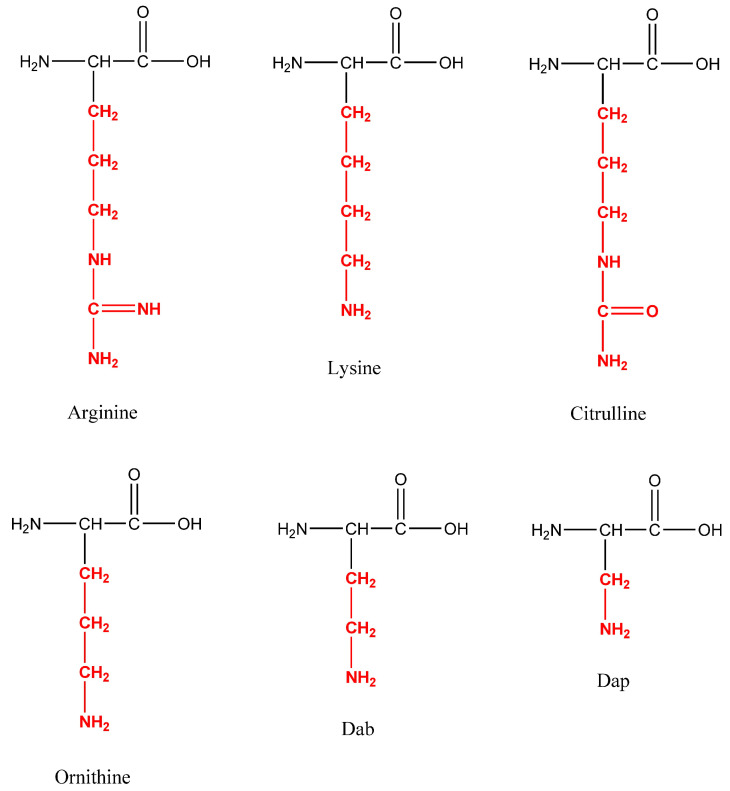
Structures of the moderated amino acids in the position 7 of Temporin A.

Temporin A, Temporin F, and novel C-terminal amidated analogs with a general structure FLPLIG-**X**-VLSGIL-NH_2_, where **X** represents Arg, Lys, Cit, Orn, Dab, and Dap, were synthesized (Figure 1). 

**Figure 2:**
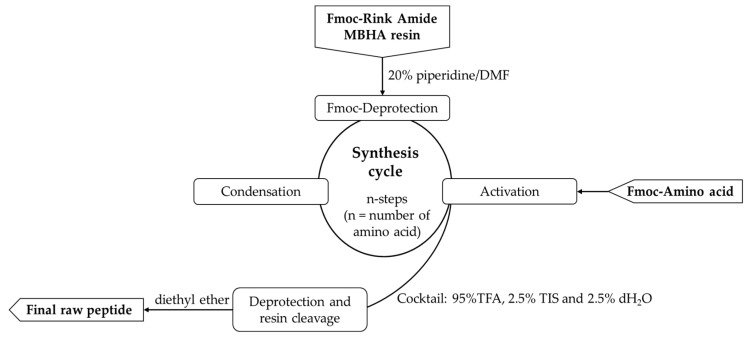
General procedure for solid-phase peptide synthesis (SPPS).

All peptides were obtained by a SPPS, Fmoc/Ot-Bu strategy following the cycle presented in Figure 2. The physicochemical characteristics of the newly synthesized molecules are presented in Table 1. 

### 3.2. Safety Testing

**Figure 3:**
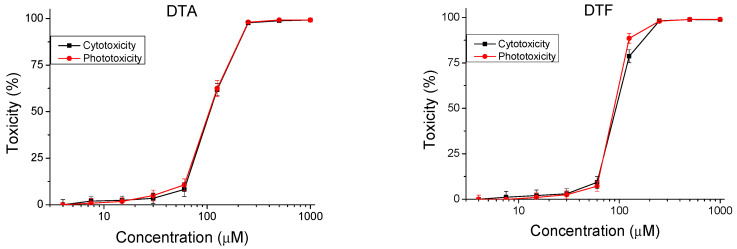

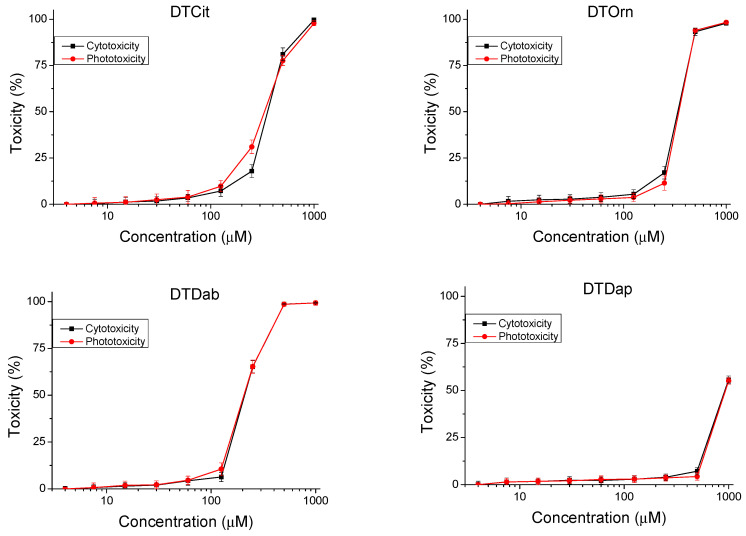
Dose–response curves for cyto- and phototoxicity of peptide analogs determined in BALB 3T3 clone A31 cell line. Values are means ± SD, *n* = 6.

The peptides were studied for safety using an in vitro 3T3 NRU test. The cytotoxicity/phototoxicity expressed in % relative to the negative control was determined. Dose–response dependence was observed for all peptide analogs (Figure 3). At a concentration of 60 µg/mL, no cytotoxic effect was observed on the test peptides. CC_50_ values (50% cytotoxic concentration) were calculated through nonlinear regression analysis (Table 2). The peptide analogs tested had significantly lower toxicity (*p* < 0.001) than the natural peptide Temporin A. The exception was DTF, where increased toxicity (CC_50_ = 92.40 ± 2.82 µM) was observed. To evaluate the phototoxic potential of the investigated peptide analogs, we used the photo irritation factor (PIF), which was calculated using the following formula: PIF = CC_50_ − Irr/CC_50_ + Irr. The PIF showed us the probability that the test peptide may cause a phototoxic effect (PIF < 2 = not phototoxic, 2 ≤ PIF < 5 = probable phototoxicity, PIF ≥ 5 = phototoxic). For all tested peptides, the calculated PIF was <2, which shows a high level of photo safety. 

### 3.3. In Vitro Antiproliferative Activity

**Figure 4:**
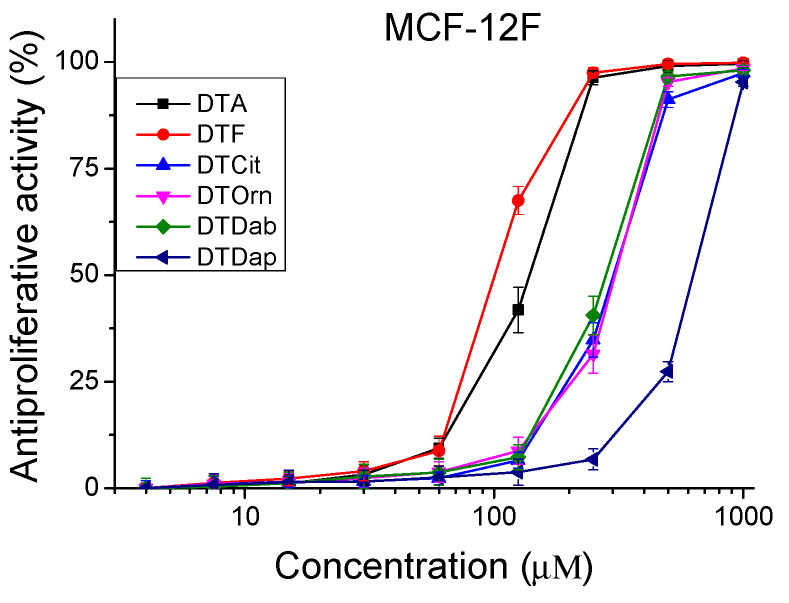
Antiproliferative activity of peptide analogs in the non-tumorigenic cell line MCF-12F (healthy tissue model). Values are means ± SD, n = 6.

**Figure 5:**
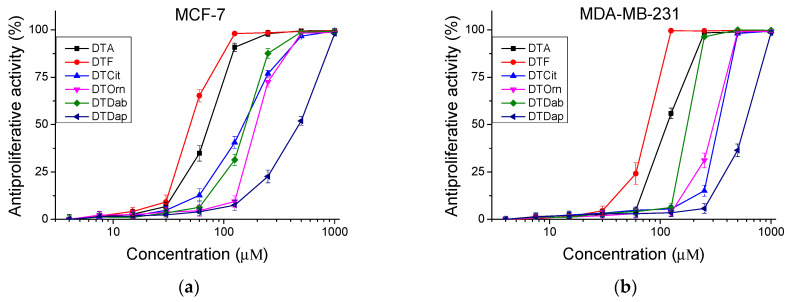
Antiproliferative activity of peptides determined in tumor cell lines (**a**) MCF-7 and (**b**) MDA-MB-231 (in vitro model of breast cancer). Values are means ± SD, *n* = 6.

The peptides were studied for their antiproliferative activity with a MTT dye-reduction assay. Cells were incubated with the test peptides at a concentration from 4 to 1000 µM under standard cell culture conditions. The antiproliferative activity expressed in % relative to the negative control was determined. The obtained results are shown in Figure 4 and Figure 5. The mean IC_50_ values and selectivity index were calculated and are presented in Table 3.

### 3.4. Antimicrobial Activity

#### 3.4.1. Disk-Diffusion Method

The disk-diffusion method was used as a preliminary method, in order to determine the susceptibility of the testing strains towards the parent peptides DTA and DTF, as well as the newly synthesized analogs. For the purpose of this investigation, the microbial cultures were placed on sterile medium plates over a thin layer (100 μL). On paper sterile discs, 20 μL of 1.4 mg/mL and 10 mg/mL peptide solutions of the test peptides were placed. Furthermore, the discs were incorporated into the sterile plates. As a positive control, a corresponding antibiotic was used. For a negative control, a 10% ethanol solution in water was used. The studies were performed in triplicate for each concentration level and the diameter of inhibition zones (IZ) in mm was representative of the average value of the three obtained values. The standard deviation (SD) was also calculated and presented. The results attained are presented in Table 4 for the concentration of 1.4 mg/mL and Table 5 for the concentration of 10 mg/mL. The formed zones around the tested peptides appeared to be about 3-times less compared to the control antibiotics according to Table 4. The yeast strain did not show any sensitivity to the investigated samples. All obtained zones can be seen in the figures presented in Table 6. 

For each synthesized analog and the parent peptides, the percent inhibition was calculated for *B. subtilis* 3562, *A. oxydans* 9333, and *P. aeruginosa* 3700 in both concentration levels, where the inhibition zone of the antibiotic was taken as a 100 percent inhibition, and from there the percent for each peptide analog was extrapolated. The values are summarized in Table 7. 

#### 3.4.2. Minimal Inhibitory Concentration

In order to determine the minimum inhibitory concentrations of the tested samples, the following analyses were performed. The microbial cultures were added to the 96-well plates with peptide solutions with concentrations of 0 to 320 µg/mL. The plates were then incubated for 24 h and the absorbance was measured at 630 nm. The studies were triplicates. The obtained MIC values are summarized in Table 8. 

Bactericidal activity was determined for *B. subtilis* 3562, *A. oxydans* 9333, and *P. aeruginosa* 3700. The parent peptide had MBC values of 320 µg/mL for *B. subtilis* 3562 and *P. aeruginosa* 3700. 

## 4. Discussion

A standard SPPS using Fmoc/O*t*-Bu strategy on Fmoc-Rink-amide MBHA solid phase carrier was employed for the synthesis of targeted Temporins A and F, as well as their analogs containing unnatural amino acids Orn, Dab, Dap, and Cit. Concerning the newly synthesized Temporin analogs containing unnatural amino acids, herein, the aim of this study was to investigate the role of the basicity of the amino acid in the 7th position of the molecule on both the antiproliferative and antimicrobial activities. 

Taking into account the results obtained from the disk-diffusion method, the newly synthesized analogs, except the one containing non-basic Cit residue in the lateral chain (DTCit), exhibited the same tendency in bacterial activity as those reported by Rommero et al. [13]. Thus, the newly synthesized Orn-, Dab-, and Dap-containing analogs were more active towards Gram-positive bacteria. However, the removal of a positive charge in the lateral chain of Cit led to a full loss of activity. According to Rosenfeld et al. and Mangoni and Shai, a possible reason for the inactivity of Temporin A towards Gram-negative bacteria is due to the fact that the peptide oligomerizes when it comes into contact with the lipopolysaccharides and the outer membrane of the cell [14,19]. 

Considering the data obtained for MIC, it can be seen that the lowest MIC for all strains was at 80 µg/mL. The peptides showed lower MIC values against the Gram-positive bacteria *B. subtilis* 3562 and *A. oxydans* 9333, which fully correlates with the data obtained by Capparelli et al. [20]. 

However, according to the literature data reported by Paduszynska et al., Temporin A showed activity against Gram-negative *P. aeruginosa*. In our study, Temporin A had better activity against the chosen test strain *P. aeruginosa* 3700 (MIC at 320 µg/mL), whereas in the investigation of Paduszynska et al., MIC against *Pseudomonas aeruginosa* ATCC 9029 was determined at 512 mg/L [21].

The parent peptide DTA and the analog with Lys (DTF) showed the same behavior, i.e., being more active against the Gram(+) bacteria *B. subtilis* 3562 and *A. oxydans* 9333, and with the increase in the peptide concentration, the inhibition capacity increased. However, DTF appeared to have a slightly better antibacterial activity, which was reflected in the increased inhibition zones. Thus, the replacement of Arg with less basic Lys residue was positive for the antibacterial activity. 

The highest antimicrobial potential of all analogs was revealed in DTDab. Taking into account the data obtained for the antibacterial activity of all analogs, it is clear that the shortening of the side chain had a beneficial effect on the antibacterial activity up to two methylene groups length in the lateral chain. Furthermore, shortening of the lateral chain led to a lack of a better antimicrobial effect. DTDap analog had no activity at low concentrations towards *A. oxydans* 9333 and *P. aeruginosa* 3700 but exhibited inhibition at higher values. However, it is interesting to mention that, concerning the other Gram(+) bacteria *B. subtilis* 3562 when using the disk-diffusion method, the same analog did not show any activity, but it had a MIC value of 320 µg/mL. 

The other Gram-negative bacteria *E. coli* 8785 was resistant to treatment with all newly synthesized analogs and no inhibition zone was formed when using the disk-diffusion method. However, herein, the MIC values for DTA, DTF, and DTDab were also monitored. This could be explained due to the differences between the two used methods for antibacterial activity monitoring. According to Mercer et al., Kunin and Edmondson, and Lehrer et al., when positively charged AMP interacts with the negatively charged constituents of the agar, disk-diffusion methods either significantly underestimate the activity or completely mask the real activity [22,23,24]. The same phenomenon was observed with the analog DTCit and the Gram(+) bacteria *A. oxydans* 9333. The analog DTOrn had a bactericidal effect at 320 µg/mL against *B. subtilis* 3562 and at 160 µg/mL against *A. oxydans* 9333. DTCit also had a MBC value of 160 µg/mL against *A. oxydans* 9333.

When looking over the obtained MIC values and taking into consideration the differences in the six amino acids, it could be concluded that a bulkier, longer, and thus more basic side chain is needed at position 7 in order to have a lower MIC value. A shorter and less basic amino acid such as Dab delivers greater inhibition zones. Furthermore, the removal of the positive charge in the lateral chain leads to a loss of the antibacterial properties evident with the analog DTCit. 

The parent peptide DTA and the previously reported DTF had antifungal activity against *C. albicans* 74, corresponding to the findings of Wade et al. [15]. The higher basicity of Arg and Lys proved to be vital for the antifungal potential of the temporin analogs.

Literature data indicated that temporins are toxic to normal mammalian cells within IC_50_ concentrations of 50 μM to 100 μM [25,26]. This is a significantly higher concentration than the concentrations at which antibacterial activity is observed [27,28]. However, methods and possibilities are being sought to modify temporins in order to increase their safety. Four of the investigated peptide analogues of Temporin A (DTCit, DTOrn, DTDab, and DTDap) showed significantly lower cytotoxicity than the original peptide (DTA). Furthermore, the photosafety test showed that the investigated peptide analogs were not phototoxic. These results show that the indicated peptides have the potential to be used safely for topical and systemic applications. 

An antiproliferative activity test was performed on human cell lines, an in vitro model of healthy tissue and two types of breast cancer. The original peptide (DTA) and that substituted in the seventh position with Lys peptide (DTP) caused the highest antiproliferative effect in the cell line MCF-7 (luminal type A breast cancer). We also observed selectivity (SI > 2) with the Citrulline-substituted peptide (DTCit). In contrast, the investigated peptide analogs did not show selectivity (SI < 2) against basal type B breast cancer (MDA-MB-231 cell line). These results support the literature data on the antitumor activity of positively charged temporins and their analogues [29,30], where the authors studied different types of tumor cell lines (A549, MCF-7, MDA-MB-231). A panel of different types tumor cell lines could also be tested for more information about their antiproliferative properties.

The investigated peptide analogues had a lower antiproliferative effect compared to Temporin A (DTA), except for DTF which had an IC_50_ = 100.48 ± 3.30 µM for the MCF-12F cell line. The lowest antiproliferative effect against all tested cell lines combined with the lowest cito- and phototoxicity was observed after treatment with the peptide analog DTDap, the analog containing the shortest side chain and with the lowest basicity. The MCF-7 cell line was significantly more sensitive to the studied series of peptide analogs than MDA-MB-231. In MCF-7 cells, the highest antiproliferative effect was caused by the parent peptides DTF and DTA, with IC_50_ = 49.75 ± 1.90 µM and 73.15 ± 3.36 µM, respectively. Significant selectivity (SI > 2) was observed for the peptides DTF and the analog containing unnatural amino acid Cit (DTCit) in the MCF-7 cell line. The same analog containing a non-basic lateral chain had a relatively low cytotoxicity and a lack of phototoxicity. In contrast, the investigated peptide analogs were not sufficiently selective against triple-negative basal type B breast cancer (MDA-MB-231). Thus, the obtained results reveal that decreasing the basicity of the lateral chain from DTA through DTF and DTCit led to better selectivity according to the MCF-7 cell line. However, in the same direction, decreasing the basicity of the lateral chain of the temporin analogs led to a reduction in their antiproliferative effect. These results strongly correlate with the data obtained by Zou et al. for AMP defensins [31].

## 5. Conclusions

This study was dedicated to an SAR study of the role of the basicity of amino acid Arg in the position 7 of Temporin A molecule in antimicrobial activity. The obtained results reveal the following:

- The Lys residue contained in the Temporin F molecule was the optimal residue for the combination of both antibacterial and antiproliferative activity;
- The optimal lateral chain length for antibacterial activity was found to be two methylene groups in the molecule of Dab residue;
- A bulkier, longer, and thus more basic side chain is needed at position 7 in order to have a lower MIC value, whereas a shorter and less basic amino acid such as Dab delivered greater inhibition zones when using the disk-diffusion method;
- The removal of positive charge in the lateral chain led to a loss of the antibacterial properties evident with the analog DTCit. However, this analog showed satisfactory antiproliferative activity combined with a relatively low cytotoxicity and lack of phototoxicity, as well as the best selectivity according to the MCF-7 cell line.

## Figures and Tables

**Table 1:** Structure and analytical data for newly synthesized compounds.

Code	Structure	Molecular Formula	MM _exact_ g/mol	[M+H]^+^ Observed g/mol	[M+Na]^+^ Observed g/mol	RT min	α_D_^20^ [°] *	M.p. [°C]
DTA	FLPLIG-R-VLSGIL-NH_2_	C_68_H_117_N_17_O_14_	1395.90	1397.00	1418.95	4.513	−38	158 ± 2
DTF	FLPLIG-K-VLSGIL-NH_2_	C_68_H_117_N_15_O_14_	1367.89	1368.80	1390.75	4.501	−40	155 ± 3
DTCit	FLPLIG-Cit-VLSGIL-NH_2_	C_68_H_116_N_16_O_15_	1396.88	1397.75	1419.75	5.793	−34	182 ± 2
DTOrn	FLPLIG-Orn-VLSGIL-NH_2_	C_67_H_115_N_15_O_14_	1353.87	1354.70	1376.70	4.364	−42	123 ± 2
DTDab	FLPLIG-Dab-VLSGIL-NH_2_	C_66_H_112_N_14_O_15_	1340.84	1340.75	1362.70	4.191	−52	138 ± 4
DTDap	FLPLIG-Dap-VLSGIL-NH_2_	C_65_H_110_N_14_O_15_	1326.83	1326.65	1348.70	4.077	−48	140 ± 1

* methanol (c = 1).

**Table 2:** Cytotoxicity/phototoxicity in BALB 3T3 clone A31 cell line, mean CC_50_ values and PIF factor.

Code	Mean CC_50_ ± SD (µM)		PIF *
	−Irr	+Irr	
DTA	106.32 ± 4.18	105.40 ± 4.88	1.01
DTF	92.40 ± 2.82	88.35 ± 1.48	1.05
DTCit	356.09 ± 7.64	331.78 ± 12.30	1.07
DTOrn	337.27 ± 7.09	345.1 ± 6.41	0.98
DTDab	209.57 ± 6.69	206.53 ± 7.41	1.01
DTDap	923.84 ± 21.56	933.3 ± 20.7	0.99

* Photo irritation factor: PIF < 2 = not phototoxic, 2 < PIF < 5 = possible phototoxicity, PIF > 5 = phototoxic.

**Table 3:** Average IC_50_ values and selectivity index.

Compounds	Mean IC_50_ ± SD (µM)			SI *	
	MCF-12F	MCF-7	MDA-MB-231	MCF-7	MDA-MB-231
DTA	138.65 ± 8.36	**73.15 ± 3.36**	115.13 ± 4.04	1.90	1.20
DTF	100.48 ± 3.30	**49.75 ± 1.90**	77.01 ± 2.92	**2.02**	1.30
DTCit	300.73 ± 10.49	149.69 ± 6.94	334.9 ± 4.37	**2.01**	0.90
DTOrn	305.59 ± 10.75	195.62 ± 3.63	302.87 ± 8.67	1.56	1.01
DTDab	280.25 ± 13.59	157.45 ± 4.97	174.91 ± 1.35	1.78	1.60
DTDap	**630.44 ± 9.97**	472.61 ± 16.21	580.97 ± 17.50	1.33	1.09

* Selectivity Index, SI = IC_50_ MCF-12F/IC_50_ tumor cells.

**Table 4:** Inhibition zones in mm for concentration level 1.4 mg/mL for all targeted peptides and refereed antibiotics.

Code	Structure	*B. subtilis* 3562	*E. coli* 8785	*A. oxydans* 9333	*P. aeruginosa* 3700	*C. albicans* 74	Chloramphenicol [30 µg/disk]	Itraconazole [10 µg/disk]
							*B. subtilis* 3562	*C. albicans* 74
DTA	FLPLIG-R-VLSGIL-NH_2_	8.8 ± 0.3	0.0	6.8 ± 0.3	7.8 ± 0.3	0.0	26.0	15.0
DTF	FLPLIG-K-VLSGIL-NH_2_	8.5 ± 0.5	0.0	6.7 ± 0.3	7.0 ± 0.5	0.0	25.0	15.0
DTCit	FLPLIG-Cit-VLSGIL-NH_2_	0.0	0.0	0.0	0.0	0.0	25.0	14.0
DTOrn	FLPLIG-Orn-VLSGIL-NH_2_	0.0	0.0	0.0	0.0	0.0	25.0	15.0
DTDab	FLPLIG-Dab-VLSGIL-NH_2_	0.0	0.0	8.2 ± 0.3	7.7 ± 0.6	0.0	25.0	14.0
DTDap	FLPLIG-Dap-VLSGIL-NH_2_	0.0	0.0	0.0	0.0	0.0	26.0	14.0

**Table 5:** Inhibition zones in mm for concentration level 10 mg/mL.

							Gentamicin [10 µg/disk]		
Code	Structure	*B. subtilis* 3562	*E. coli* 8785	*A. oxydans* 9333	*P. aeruginosa* 3700	*C. albicans* 74	*E. coli* 8785	*A. oxydans* 9333	*P. aeruginosa* 3700
DTA	FLPLIG-R-VLSGIL-NH_2_	8.8 ± 0.8	0.0	9.7 ± 0.6	9.3 ± 0.6	0.0	18.0	18.0	17.0
DTF	FLPLIG-K-VLSGIL-NH_2_	12.7 ± 0.6	0.0	11.5 ± 0.5	10.3 ± 0.8	0.0	18.0	20.0	16.0
DTCit	FLPLIG-Cit-VLSGIL-NH_2_	0.0	0.0	0.0	0.0	0.0	18.0	19.0	16.0
DTOrn	FLPLIG-Orn-VLSGIL-NH_2_	8.2 ± 0.3	0.0	8.3 ± 0.6	11.3 ± 0.6	0.0	18.0	19.0	16.0
DTDab	FLPLIG-Dab-VLSGIL-NH_2_	11.3 ± 0.6	0.0	14.5 ± 0.5	14.3 ± 0.6	0.0	18.0	19.0	16.0
DTDap	FLPLIG-Dap-VLSGIL-NH_2_	0.0	0.0	9.8 ± 0.3	8.8 ± 0.8	0.0	18.0	18.5	17.0

**Table 6:** Antimicrobial test results obtained by disk-diffusion method.

	Scheme of Experiment	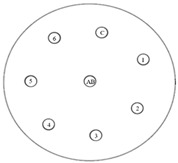	Positions 1–3: 1.4 mg/mL Positions 4–6: 10 mg/mL AB: Antibiotic C: Control (10% EtOH/H_2_O)		
	***B. subtilis* 3562**	***E. coli* 8785**	***A. oxydans* 9333**	***P. aeruginosa* 3700**	***C. albicans* 74**
**DTA**	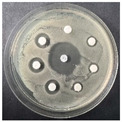	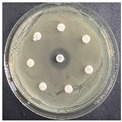	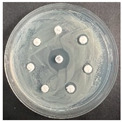	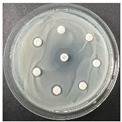	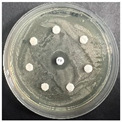
**DTF**	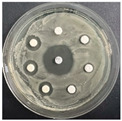	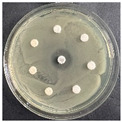	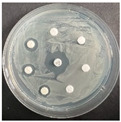	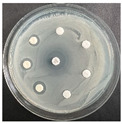	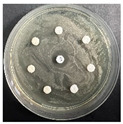
**DTCit**	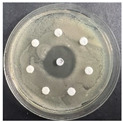	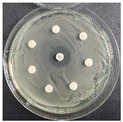	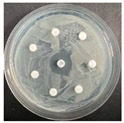	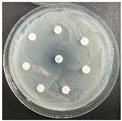	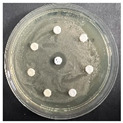
**DTOrn**	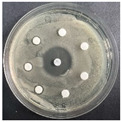	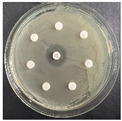	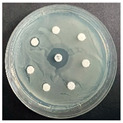	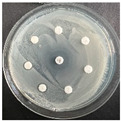	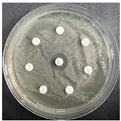
**DTDab**	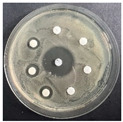	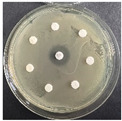	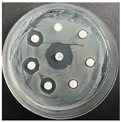	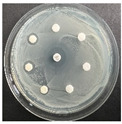	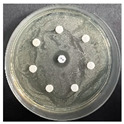
**DTDap**	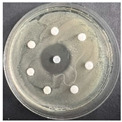	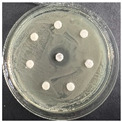	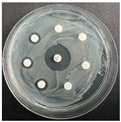	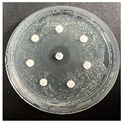	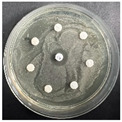

**Table 7:** Inhibition effect of peptide analogs against test microorganisms [%].

Strain		*B. subtilis* 3562		*A. oxydans* 9333		*P. aeruginosa* 3700	
	Concentration	1.4 mg/mL	10 mg/mL	1.4 mg/mL	10 mg/mL	1.4 mg/mL	10 mg/mL
Code							
DTA		33.8	33.8	37.8	53.9	45.9	54.7
DTF		34.0	50.8	33.5	57.5	43.8	64.4
DTCit		0.0	0.0	0.0	0.0	0.0	0.0
DTOrn		0.0	32.8	0.0	43.7	0.0	70.6
DTDab		0.0	45.2	43.2	76.3	48.1	89.4
DTDap		0.0	0.0	0.0	53.0	0.0	51.8

**Table 8:** MIC values of Temporin A and novel analogs [µg/mL].

Code	Structure	*B. subtilis* 3562	*E. coli* 8785	*A. oxydans* 9333	*P. aeruginosa* 3700	*C. albicans* 74
DTA	FLPLIG-R-VLSGIL-NH_2_	80 µg/mL	320 µg/mL	80 µg/mL	320 µg/mL	320 µg/mL
DTF	FLPLIG-K-VLSGIL-NH_2_	80 µg/mL	320 µg/mL	80 µg/mL	160 µg/mL	320 µg/mL
DTCit	FLPLIG-Cit-VLSGIL-NH_2_	NI	NI	160 µg/mL	NI	NI
DTOrn	FLPLIG-Orn-VLSGIL-NH_2_	160 µg/mL	NI	160 µg/mL	NI	NI
DTDab	FLPLIG-Dab-VLSGIL-NH_2_	160 µg/mL	320 µg/mL	320 µg/mL	320 µg/mL	NI
DTDap	FLPLIG-Dap-VLSGIL-NH_2_	320 µg/mL	NI	NI	NI	NI

Note: NI—no inhibition monitored.

## Data Availability

Small quantities of all substances are available from authors from the University of Chemical Technology and Metallurgy.
